# Intraoperative and Postoperative Complications of Laser *in situ* Keratomileusis Flap Creation Using IntraLase Femtosecond Laser and Mechanical Microkeratomes

**DOI:** 10.4103/0974-9233.61217

**Published:** 2010

**Authors:** Ladan Espandar, Jay Meyer

**Affiliations:** Department of Ophthalmology, Tulane University, New Orleans, LA, USA; 1 North Carolina University, Chapel Hill, NC, USA

**Keywords:** IntraLase Femtosecond Microkeratome, Laser *in situ* Keratomileusis Flap, Mechanical Microkeratome

## Abstract

An essential step of laser *in situ* keratomileusis surgery is corneal flap creation, Femtosecond (FS)-assisted or mechanical microkeratome. Each type has rare intraoperative and postoperative complication rates. Several recent studies have identified risk factors and guidelines to help manage these complications. Fortunately, studies have shown no loss of best-corrected visual acuity (BCVA) after the management of intraoperative and postoperative complications in IntraLase FS and mechanical microkeratome. Refractive surgeons need to be aware of the types of complications that can occur, how to avoid them and how to manage them to ensure the best possible outcomes.

## INTRODUCTION

Laser *in situ* keratomileusis (LASIK)is currently the most popular refractive surgery procedure worldwide.A critical step of LASIK surgery is the creation of the corneal flap. The two most common ways to create the flap are with a femtosecond (FS) laser or mechanical microkeratome. In recent years, the IntraLase FS laser (IntraLase Corp., Santa Ana, CA, USA), has gained increasing popularity.^1^ However, the majority of refractive surgeons are still using the mechanical microkeratome. The purpose of this review is to compare and contrast the various types and rates of complications when using these two methods of flap creation.

## MECHANICAL MICROKERATOME

The mechanical microkeratome uses shear force traveling across the corneal stroma with an oscillating blade to create a flap. Several types of mechanical microkeratomes are available, such as the Amadeus (Advanced Medical Optics, Santa Ana, CA, USA), Hansatome (Technolas Bausch and Lomb, Rochester, NY, USA), Summit-Krumeich-Barraquer (SKBM; Alcon, Ft Worth, TX, USA), Supratome (Schwind, Kleinostheim, Germany) and Automated Corneal Shaper (Chiron Vision Corp., Emeryville, CA, USA). The differences between the various models include the number of motors, single-handed versus double-handed keratome operation, flap diameter, flap thickness and cutting rate related to the oscillation rate of the knife motor.

Jacobs and Taravella,^2^ in a retrospective analysis of 28,530 primary LASIK cases, reported a 0.302% total intraoperative complication rate using the Automated Corneal Shaper and Hansatome microkeratomes. Complications included failure to achieve the appropriate intraocular pressure (0.034%), partial flaps (0.099%), buttonholes(0.070%), thin or irregular flaps (0.087%) and free flaps (0.012%).The Hansatome microkeratome was associated with a lower complication rate (0.16%) than the Automated Corneal Shaper (6.38%) (*P* < 0.005).

Nakano *et al*.^3^ also showed that different microkeratomes have statistically different intraoperative complication rates, with a higher rate seen in the Automated Corneal Shaper (1.26%) relative to the Hansatome and MK-2000 (Nidek Inc., Fremont, CA, USA) (0.63% each). Complications included incomplete flaps (0.23%), buttonholes (0.13%), thin flaps (0.08%) and free flaps (0.08%).

One main step in the prevention of flap complications is risk-factor identification and modification. Flap thickness created by the mechanical microkeratome can be a major variable according to some studies. Yau and Cheng^4^ argued that even with the same microkeratome (Moria M2, Doylestown, PA, USA), blades from different manufacturers produce significantly different flap thickness. However, Alio and Penero^5^ showed that the Moria M2 and Carrizo-Pendular (SCHWIND eye-tech-solutions, Kleinostheim, Germany) microkeratomes created predictable flap thickness, as measured by very-high frequency digital ultrasound.

Epithelial defect formation during LASIK using a mechanical microkeratome has been studied for risk factor association. One study found increasing patient age (especially over 40 years) and preoperative hyperopia as risk factors for epithelial defect formation with the Hansatome microkeratome.^6^ Another review of 1,873 eyes that underwent LASIK with the Automated Corneal Shaper microkeratome showed that the risk of epithelial damage was associated with increasing age, years of contact lens wear and intraoperative epithelial damage in the first eye during simultaneous bilateral LASIK.^7^

Preoperative keratometric power has been found to affect intraoperative complications as well. From areview of 34,099 eyes, flatter corneas tended to have more free caps and incomplete flaps whereas eyes with steeper corneas tended to have more epithelial abrasions and thin or irregular flaps.^8^ Preoperative risk assessment should not be limited to the cornea. Asano-Kato *et al*.^9^ found that narrow palpebral fissures (common in Asian populations) might be a risk factor for insufficient fixation of a microkeratome.

## INTRALASE FEMTOSECOND LASER MICROKERATOME

The IntraLase FS laser is a solid-state laser used to create corneal lamellar flaps by producing a circular cleavage plane starting at one side and progressing across the cornea in a back and forth pattern. It applies patterned pulses of ultrashort wavelength energy at many intrastromal points with a predetermined depth. Each laser pulse generates a small amount of microplasma, which results in microscopic gas bubbles in the interface and creates the flap. It creates a flap edge of a programmable angle by using a circumferential pattern of progressively shallower pulses. A predefined arc along the edge is left uncut to create the hinge. The entire process takes place through a glass applanation plate that is fixed to the eye with a low-pressure suction ring. Creation of a smooth optical interface has been the primary goal of flap production in order to have better optical quality, reduced aberrations and improved visual outcomes. Sarayaba *et al*.,^10^ in a cadaveric study, evaluated the stromal bed quality produced by the Hansatome microkeratome with a 160-µm head and IntraLase 15- and 30-kHz FS laser with 110 µm thickness by scanning electron microscopy. They found that the 30-kHz IntraLase created a smoother stromal bed compared with the 15-kHz and Hansatome due to a tighter spot/line separation and lower energy per pulse.

The accuracy of the LASIK flap thickness is a key risk factor for flap complications and ectasia following LASIK. Kezirian and Stonecipher,^11^ in a retrospective study, showed that IntraLase produced a more predictable flap relative to a mechanical microkeratome as measured by a DGH Pachette 50/60 kHz pachymeter. Binder^12^ showed predictability of flap thickness, flap diameter and hinge location, which eliminates the risk for cap perforations. Kim *et al*.^13^ showed a highly reproducible flap thickness with IntraLase, as measured by optical coherence tomography (Visante, Carl Zeiss Meditec).

Several studies have compared the visual outcomes of LASIK using the IntraLase versus mechanical microkeratome for creation of the flap. Durrie and Kezirian^14^ showed in a prospective contralateral-eye study that the IntraLase group had significantly better mean uncorrected visual acuity (UCVA), less residual astigmatism and fewer trefoils in aberrometry. However, Patel *et al*.,^15^ in a randomized, controlled, paired-eye study, reported 6% higher corneal backscatter (haziness examined by confocal microscopy) in IntraLase flaps relative to mechanical at 1 month, but not at 3 or 6 months postoperatively. No difference was found in high-contrast visual acuity and contrast sensitivity. Therefore, the method of flap creation did not affect visual outcomes in this study. In a more recent retrospective study of 2,000 eyes, the percentage of eyes that achieved an UCVA of 20/20or better was significantly higher in the FS laser relative to mechanical microkeratome and a lower percentage of eyes in the FS laser group lost two or more lines of BSCVA postoperatively.^16^

Even though the IntraLase FS laser may offer several advantages compared to the mechanical microkeratome, complications with the FS laser have been reported in the literature. According to several published reports, the incidence of diffuse lamellar keratitis (DLK) is greater in eyes where the LASIK flap was created with a FS laser compared to those created with a mechanical microkeratome.^17^–^19^ Gas breakthrough,^20^ opaque bubble layer,^18^ suction loss leading to incompleteflap^12^ and transient light sensitivity syndrome(TLSS)^21^ have also been reported with the use of the FS laser.

Haft *et al*.,^22^ in a retrospective, noncomparative, interventional case series, described intra- and postoperative complications of the IntraLase FS microkeratome in 4,772 eyes. They reported a total complication rate of 0.92%. Intraoperative (flap-related) complications developed in 0.25%, premature breakthrough of gas through the epithelium within the flap margins was seen in 0.17%, incomplete flap due to suction loss was found in 0.06% and one eye had an irregular flap due to a previous scar. For premature breakthrough gas, no management was suggested and one might continue the procedure without further complications. For incomplete flaps, a second laser pass at the same level simultaneously might be performed without further complications. Postoperative complications comprised of TLSS in 0.25% and DLK (stage 1-2) in 0.42%, which was treated with an intensive course of topical steroids. Overall, none of the complications caused loss of BSCVA in any of the studied eyes.

Moshirfar *et al*., in an unpublished retrospective, interventional case series, compared the intra- and postoperative flap complication rate of the Hansatome microkeratome (896 eyes) with IntraLase FS60 laser (902 eyes). They found a 14.2% total complication rate in the Hansatome group relative to 15.2% in the IntraLase group (*P* = 0.5437). Intraoperative complications included major epithelial defect/sloughing, incomplete flap, buttonhole or vertical gas breakthrough (in the case of the FS laser), torn flap, severely decentered flap preventing ablation and gas bubble in the anterior chamber. Postoperative complications occurring within 6 weeks of the original procedure included dislocated flap, epithelial in growth (after original surgery without enhancement), DLK stages I-III, central toxic keratopathy and TLSS. The intraoperative flap complication rate was 5.3% for Hansatome and 2.9% for IntraLase (*P* = 0.0111). The most common intraoperative complication in the Hansatome group was major epithelial defect/sloughing at a rate of 2.6%, which was significantly higher than the IntraLase group (*P* = 0.0006).

When comparing buttonhole formation, those that occurred in the setting of the microkeratome were typically central and round and approximately 3-4 mm in diameter. The subepithelial gas breakthrough seen with the FS laser-created flap was often peripheral and 1-2 mm in diameter. Although gas breakthrough is not typically classified as a buttonhole, the end results were the same. The occurrence of buttonholes with the use of FS is rare,^20^ and the lower incidence of buttonholes in the FS group could be due to the fact that FS laser creates a planar flap as opposed to the meniscus-shaped flap created by the microkeratome.^12^,^23^

The incidence of torn flaps was similar in both the IntraLase (0.4%) and the Hansatome groups (0.4%). However, all flap tears in the IntraLase group occurred at the flap hinge as opposed to the more central Hansatome flap tears. An advantage of tearing at the hinge is the avoidance of the central axis and having the option of proceeding with stromal ablation the same day.

Decentered flaps have been reported as a complication with mechanical microkeratomes. A low number of flap dislocations with the FS laser are most likely due to the FS side cut's steeper angle and deeper gutter as well as the increased adhesion strength of the FS flap.^24^–^27^

The postoperative flap complication rate was 8.9% for Hansatome and 12.3% for IntraLase (*P* = 0.0201). The most common postoperative complication was DLK in both groups, 6.0% with the Hansatome and 10.6% with IntraLase (*P* = 0.0002). It has been hypothesized that accumulation of gas bubbles and the energy of the FS laser could lead to an increased inflammatory response in individuals who might be more susceptible to DLK.^17^ Even though the IntraLase group had a higher incidence of DLK, the patients did not progress to DLK stage III, and DLK can often be managed with an intense course of topical corticosteroids.

Bubbles in the anterior chamber, TLSS and rainbow glare are complications unique to the FS laser. Two case reports found that anterior chamber bubbles can interfere with pupillary tracking, but are self-limiting and resolve over a short period of time.^28^,^29^ Patients with TLSS present approximately 2-6 weeks after uneventful LASIK with severe photosensitivity.^21^ These patients have good UCVA and no inflammatory findings on slitlamp exam, and treatment with topical steroids has been found to be effective. Rainbow glare is an optical side-effect in which patients report seeing a spectrum of colored bands radiating from a white-light source when viewed in adark environment, such as a night-time setting.^30^ The exact cause of TLSS and rainbow is not known. For TLSS, it is theorized that increased energy of the FS laser can stimulate local keratocytes and/or corneal nerve endings.^21^ Stonecipher *et al*. looked at different groups showing a TLSS incidence rate of 1.0-1.4%, and noted that when the raster and side-cut energy settings were lowered (by an average of 24% and 33%, respectively), a significant reduction in the incidence of TLSS was achieved.^21^ Similar to TLSS, rainbow glare appears to occur more often with higher raster energy settings.^30^

It is noteworthy to mention that none of the studied patients with intraoperative or postoperative flap complications in IntraLase FS and mechanical microkeratome groups experienced loss of BSCVA after the management of complications.

## CONCLUSION

Microkeratome flap complications can occur with either mechanical or IntraLase FS laser created flaps. Fortunately, these complications are rare and several recent studies have identified risk factors and guidelines to help manage these complications. Prospective comparisons of mechanical and FS laser microkeratome in LASIK have shown no significant clinical differences in final visual acuity after the management of complications. Refractive surgeons need to be aware of the types of complications that can occur, how to avoid them and how to manage them to ensure the best possible outcomes.^31^
